# Supplementary material for: Comprehensive Evaluation of Different TiO2-Based Phosphopeptide Enrichment and Fractionation Methods for Phosphoproteomics
Source: Cells. 2022 Jun 28;11(13):2047. doi: 10.3390/cells11132047 (PMC9265536; doi:10.3390/cells11132047)
Supplement: Supplementary file 1 [file cells-11-02047-s001.zip › Supplemental Data Comprehensive evaluation of different TiO2-based phosphopeptide enrichment and fractionation methods for phosphoproteomics.pdf]

Supporting Information

Comprehensive evaluation of different TiO_2-based phosphopeptide enrichment and fractionation methods for phosphoproteomics

Jiaran Li[1,2,3], Jifeng Wang[1], Yumeng Yan[1,2,3], Na Li[1], Xiaoqing Qing[1,2], Ailikemu·Tuerxun[1,2,4], Xiaojing Guo[1], Xiulan Chen[1,2,3]*, Fuquan Yang[1,2,3]*

1 Key Laboratory of Protein and Peptide Pharmaceuticals & Laboratory of Proteomics, Institute of Biophysics, Chinese Academy of Sciences, Beijing 100101, China

2 University of Chinese Academy of Sciences, Beijing 100149, China

3 Sino-Danish College of University of Chinese Academy of Sciences, Beijing, 101408, China

Supplementary Materials

Supplementary Figure S1: Heatmap analysis and the number of phosphopeptides identified with the four TiO_2-based phosphopeptide enrichment methods.

Supplementary Figure S2: The physicochemical characteristics of phosphopeptides and non-phosphopeptides identified with the four phosphopeptide enrichment methods.

Supplementary Figure S3: The physicochemical characteristics of phosphopeptides identified in both TiO_2-TiO_2 and TiO_2-TiO_2-FT, and phosphopeptides exclusively identified in TiO_2-TiO_2-FT.

Supplementary Figure S4: Deamidation of phosphopeptides and non-phosphopeptides.

Supplementary Figure S5: Evaluation of MS data after the TEA-based HpH-RP fractionation.

Supplementary Table S1: Phosphopeptides identified from samples in which different amounts of peptides are used for enrichment.

Supplementary Table S2: Phosphopeptides identified from the glutamic acid method (three replicates).

Supplementary Table S3: Phosphopeptides identified from the lactic acid method (three replicates).

Supplementary Table S4: Phosphopeptides identified from the glycolic acid method (three replicates).

Supplementary Table S5: Phosphopeptides identified from the DHB method (three replicates).

Supplementary Table S6: Phosphopeptides identified from four phosphopeptide enrichment methods (combined results of three replicates of each method).

Supplementary Table S7: Peptides identified from 293T cell lysate (three replicates).

Supplementary Table S8: Phosphopeptides identified from four modified protocols of glycolic acid method (three replicates for each protocol).

Supplementary Table S9: Phosphopeptides identified from TiO_2-TiO_2-FT of double TiO_2 enrichment (three replicates and combined results).

Supplementary Table S10: Phosphopeptides identified with the glutamic acid method from 1 mg peptides (single-shot LC-MS/MS analysis of three replicates).

Supplementary Table S11: Phosphopeptides identified with the lactic acid method from 1 mg peptides (single-shot LC-MS/MS analysis of three replicates).

Supplementary Table S12: Phosphopeptides identified from the ammonia-based HpH-RP fractionation (three replicates).

Supplementary Table S13: Phosphopeptides identified from the TEA-based HpH-RP fractionation (three replicates).

Supplementary Table S14: Phosphopeptides identified from three replicates of the TEA-based HpH-RP fractionation (combined data).

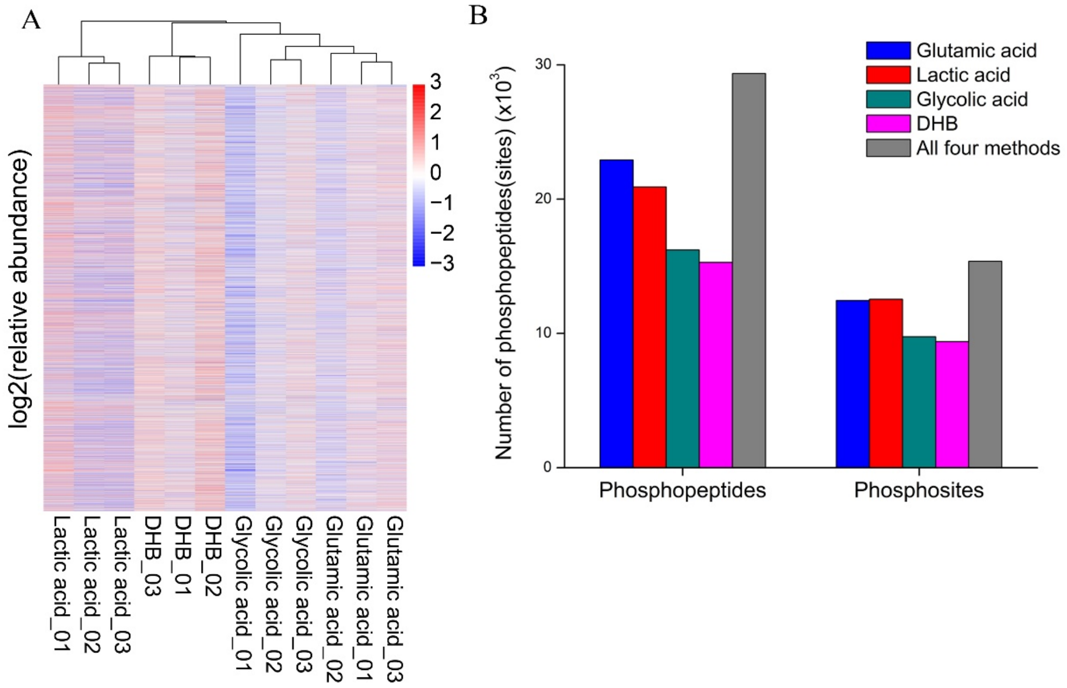

Supplementary Figure S1. Heatmap analysis and the number of phosphopeptides identified with the four TiO_2-based phosphopeptide enrichment methods. (A) Heatmap of phosphopeptides identified with the four phosphopeptide enrichment methods. The normalized intensities of quantified phosphopeptides from all samples are log2 transformed and heatmap analysis is performed using pheatmap function in the R package. **(B)** The number of phosphopeptides and phosphosites identified in each method by combining three replicates.

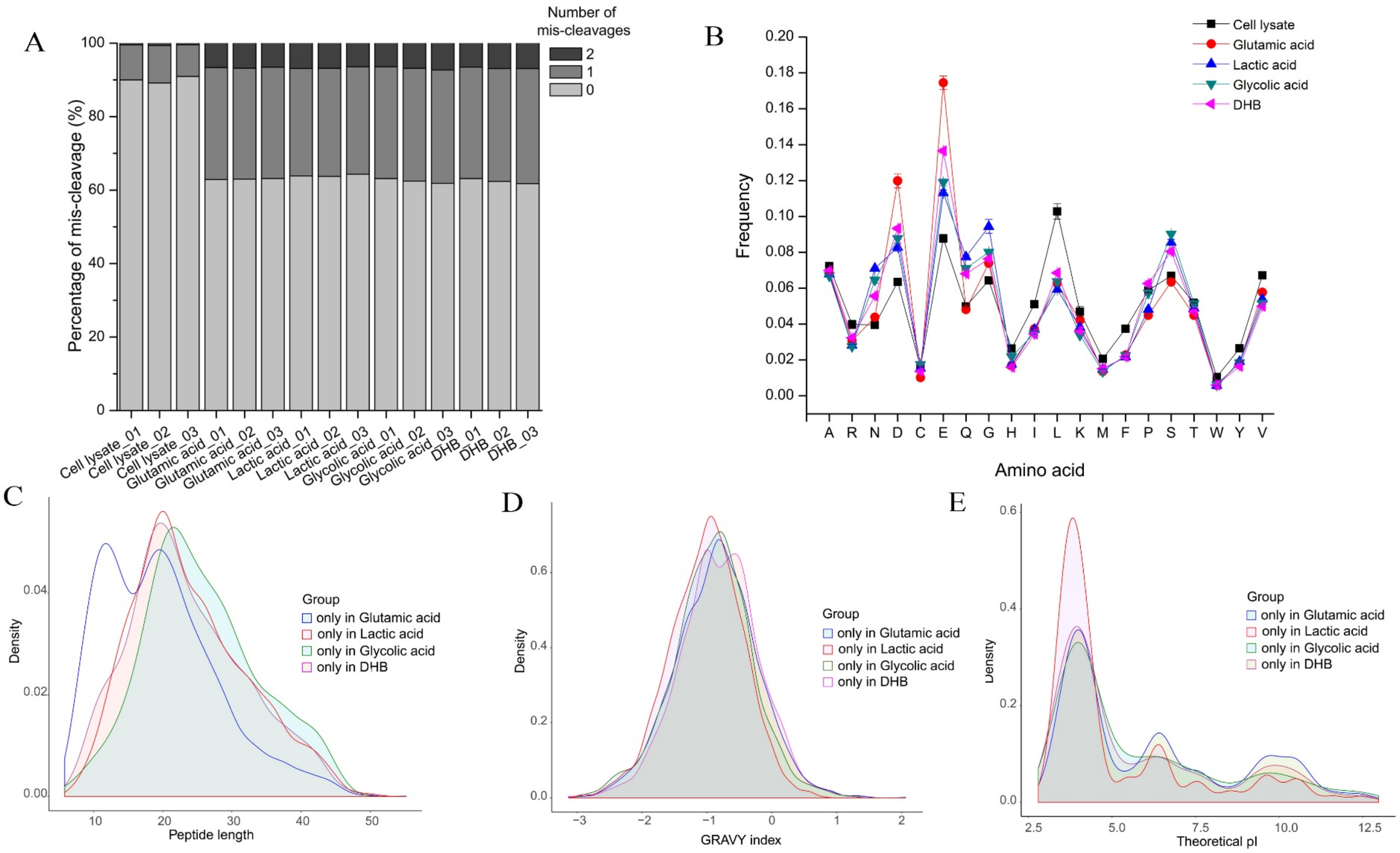
A
Number of mis-cleavages
2
1
0
Percentage of mis-cleavage (%)
Cell lysate_01
Cell lysate_02
Cell lysate_03
Glutamic acid_01
Glutamic acid_02
Glutamic acid_03
Lactic acid_01
Lactic acid_02
Lactic acid_03
Glycolic acid_01
Glycolic acid_02
Glycolic acid_03
DHB_01
DHB_02
DHB_03
B
Cell lysate
Glutamic acid
Lactic acid
Glycolic acid
DHB
Frequency
Amino acid
A R N D C E Q G H I L K M F P S T W Y V
C
Group
only in Glutamic acid
only in Lactic acid
only in Glycolic acid
only in DHB
Density
Peptide length
D
Group
only in Glutamic acid
only in Lactic acid
only in Glycolic acid
only in DHB
Density
GRAVY index
E
Group
only in Glutamic acid
only in Lactic acid
only in Glycolic acid
only in DHB
Density
Theoretical pI

Supplementary Figure S2. The physicochemical characteristics of phosphopeptides and non-phosphopeptides identified by the four phosphopeptide enrichment methods. (A) The percentage of mis-cleavages in peptides from cell lysate and phosphopeptides identified by the four phosphopeptide enrichment methods. Three replicates are performed for each sample. **(B)** The amino acid composition of peptides identified from cell lysate and non-phosphopeptides with the four phosphopeptide enrichment methods. **(C)** The distribution of peptide length of phosphopeptides exclusively identified in the four phosphopeptide enrichment methods. **(D)** The distribution of GRAVY index of phosphopeptides exclusively identified in the four phosphopeptide enrichment methods. **(E)** The distribution of theoretical pI of phosphopeptides exclusively identified in the four phosphopeptide enrichment methods.

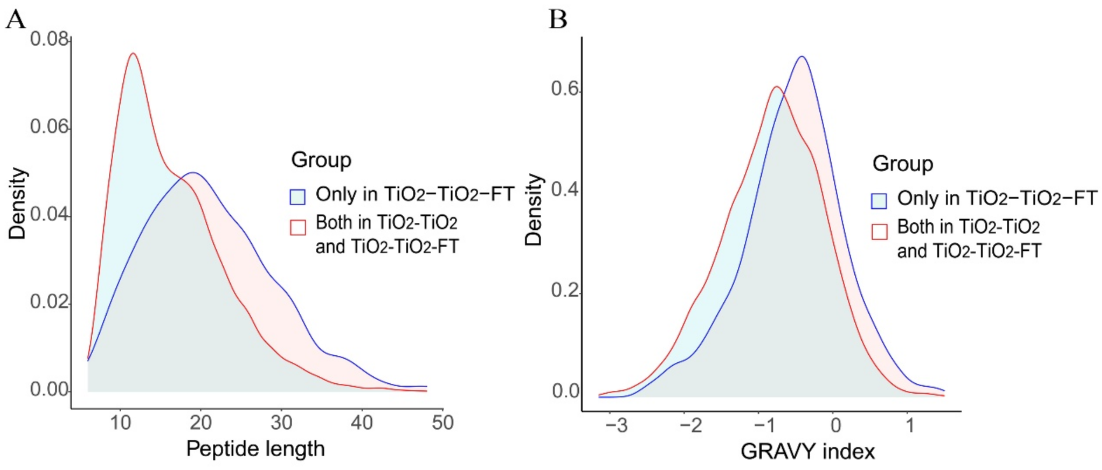

Supplementary Figure S3. The physicochemical characteristics of phosphopeptides identified in both TiO_2-TiO_2 and TiO_2-TiO_2-FT, and phosphopeptides exclusively identified in TiO_2-TiO_2-FT. (A) The distribution of length of phosphopeptides identified in both TiO_2-TiO_2 and TiO_2-TiO_2-FT, and phosphopeptides exclusively identified in TiO_2-TiO_2-FT. **(B)** The distribution of GRAVY index of phosphopeptides identified both in TiO_2-TiO_2 and TiO_2-TiO_2-FT, and phosphopeptides exclusively identified in TiO_2-TiO_2-FT.

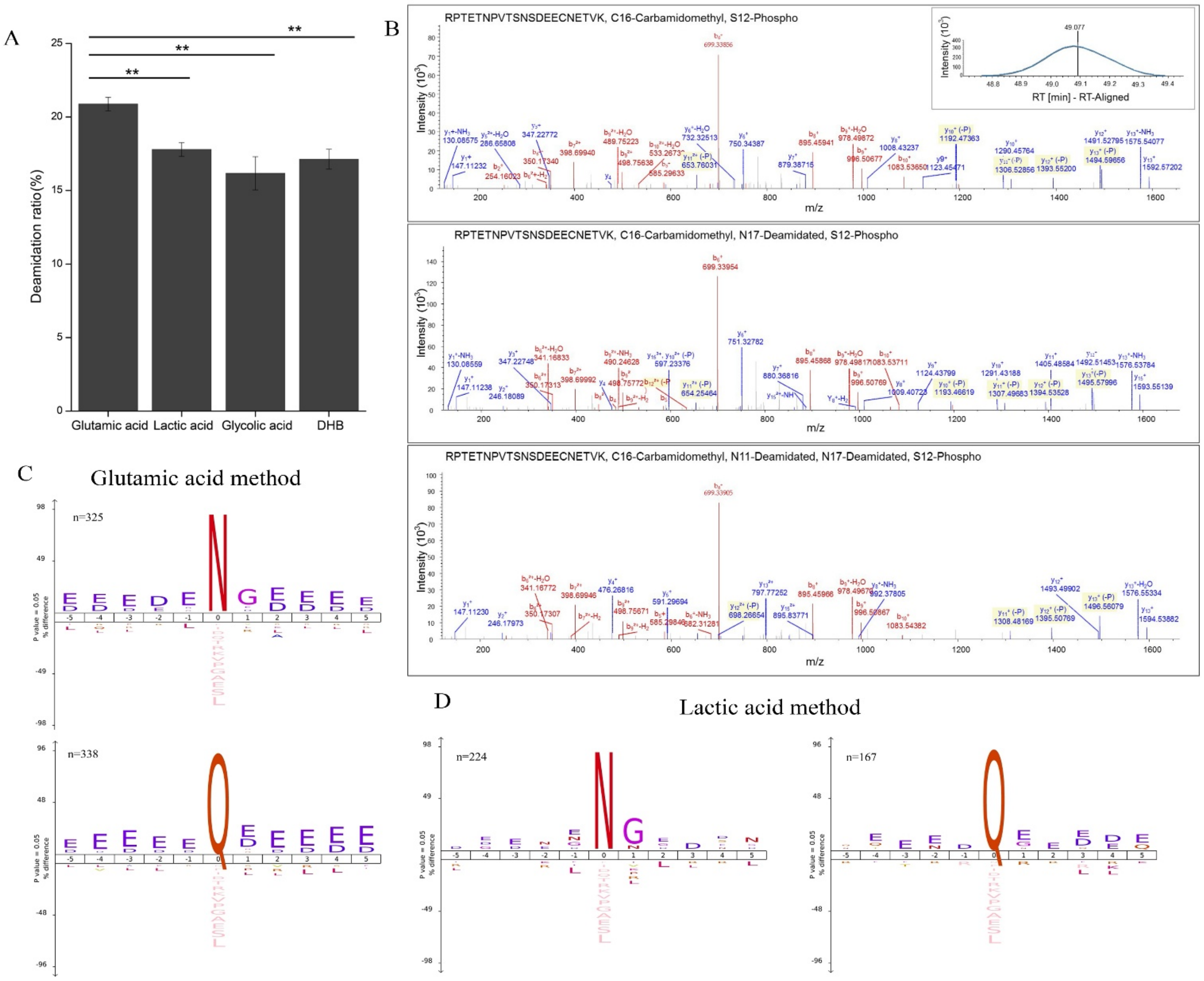
A
Deamidation ratio (%)
**
Glutamic acid
Lactic acid
Glycolic acid
DHB
B
RPTETNPVTSNSDEECNETVK, C16-Carbamidomethyl, S12-Phospho
RPTETNPVTSNSDEECNETVK, C16-Carbamidomethyl, N17-Deamidated, S12-Phospho
RPTETNPVTSNSDEECNETVK, C16-Carbamidomethyl, N11-Deamidated, N17-Deamidated, S12-Phospho
Intensity (10³)
m/z
RT [min] - RT-Aligned
C
Glutamic acid method
n=325
n=338
P value = 0.05
% difference
D
Lactic acid method
n=224
n=167

Supplementary Figure S4. Deamidation of phosphopeptides and non-phosphopeptides. **(A)** The deamidation ratio of phosphopeptides in the four phosphopeptide enrichment methods. Bars show mean± SD of the three replicates; ** $p<0.01$ (one-way ANOVA with LSD post hoc test). **(B)** MS2 spectra and retention time of phosphopeptide and their deamidated counterparts. Upper: the spectra and retention time of a phosphopeptide. Middle: the spectra and retention time of the phosphopeptide with one deamidation site. Bottom: the spectra and retention time of the phosphopeptide with two deamidation sites. (**C–D)** IceLogo sequence motif analysis of residues flanking deamidation sites in non-phosphopeptides identified in the glutamic acid method (C) and the lactic acid method (D) ($p<0.05$). The height of amino acid letters corresponds to percentage.

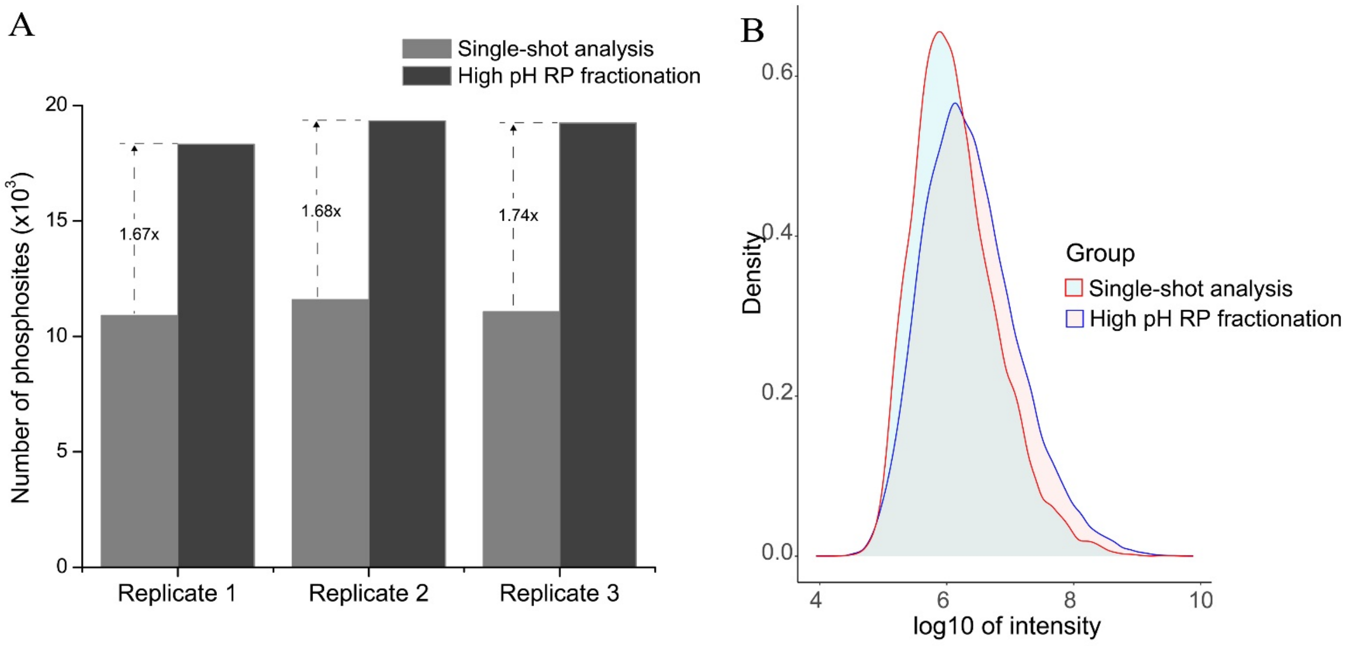

Supplementary Figure S5: Evaluation of MS data after the TEA-based HpH-RP fractionation. **(A)** The number of localized phosphopeptides in single-shot LC-MS/MS analysis and after HpH-RP fractionation. **(B)** Dynamic MS-signals range of phosphopeptides in single-shot analysis and after HpH-RP fractionation.
